# Proposed NT-ProBNP Threshold for Predicting 2-Year Heart Failure Mortality and Implications for Long-Term Community Follow-Up

**DOI:** 10.3390/epidemiologia6040059

**Published:** 2025-10-02

**Authors:** Ioana Camelia Teleanu, Gabriel Cristian Bejan, Ioana Ruxandra Poiană, Anca Mîrșu-Păun, Silviu Ionel Dumitrescu, Ana Maria Alexandra Stănescu

**Affiliations:** 1 Department of Family Medicine, “Carol Davila” University of Medicine and Pharmacy, 020021 Bucharest, Romania; ioana.fotino@drd.umfcd.ro (I.C.T.); ioana.poiana@drd.umfcd.ro (I.R.P.); alexandra.stanescu@umfcd.ro (A.M.A.S.); 2 “Carol Davila” Central Military Emergency University Hospital, 115241 Bucharest, Romania; silviu.dumitrescu@scumc.ro; 3 Faculty of Medicine, ‘Titu Maiorescu’ University, 040441 Bucharest, Romania; 4 Academy of Romanian Scientists (AOSR), 050091 Bucharest, Romania; 5 “Emil Palade” Center of Excellence for Young Researchers EP-CEYR The Academy of Romanian Scientists, 030167 Bucharest, Romania

**Keywords:** heart failure, NT-proBNP cut-off, mortality, prevention, longitudinal study

## Abstract

Background/Objectives: Pre-discharge NT-proBNP levels may serve as a helpful tool in the algorithm of assessing the long-term risk of mortality after a hospitalization for symptomatic heart failure (HF). The goals were: (a) to identify a cut-off for NT-proBNP concentrations for predicting the two-year all-cause mortality in our sample of patients, and (b) to identify risk factors associated with NT-proBNP concentrations being higher than this cut-off. Methods: The present prospective study included 96 patients diagnosed with symptomatic HF with left ventricular ejection fraction (LVEF) < 50%, who were followed for up to 2 years post-hospital discharge. Results: Levels of pre-discharge NT-proBNP were found to be predictive of all-cause mortality. We determined that an NT-proBNP cut-off score of 8700 pg/mL may predict with 75.8% sensitivity and 70.1% specificity a 4.6-fold increase in mortality risk over a period of two years in our study sample, 95% CI (2–10.8), *p* = 0.001. Predictors of NT-proBNP concentrations > 8700 pg/mL included: older age, OR 4.73, 95% CI (1.74–12.85), *p* = 0.002; lack of angiotensin converting enzyme inhibitor (ACE-I) treatment, OR 0.3, 95% CI (0.12–0.74), *p* = 0.009; low systolic blood pressure (SBP) at admission, OR 3.4, 95% CI (1.36–8.49), *p* = 0.009; and low serum hemoglobin at admission, OR 3.2, 95% CI (1.38–7.46), *p* = 0.007. Conclusions: NT-proBNP may serve as a helpful tool for predicting mortality after an episode of HF decompensation, thus allowing the implementation of appropriate long-term monitoring and treatment. Particular attention should be paid to older patients without ACE-I medication, who had SBP < 120 mmHg at admission, and/or low levels of serum hemoglobin—as these patients are more likely to have pre-discharge NT-proBNP concentrations higher than the cut-off. These findings have implications for the long-term community follow-up of patients with HF.

## 1. Introduction

Acute heart failure (AHF) represents one of the most common reasons for hospital admissions, which is unfortunately associated with a high risk of mortality—both in-hospital and long-term [1,2]. Consequently, reliable estimates of post-discharge individualized risk are stringently needed, in order to formulate effective, patient-focused long-term treatment plans that may prevent future negative outcomes [3].

N-terminal B-type natriuretic peptide (NT-proBNP) is a hormone mainly produced by ventricular myocytes in response to ventricular wall stretch determined by volume overload. It is often interpreted as a marker of congestion [4] and, as such, is used as part of the HF diagnostic algorithm (per ESC 2021 guidelines) [5]. Additionally, several trials investigated the use of NT-proBNP as a biomarker for long-term prognostic—yet reliable cut-off scores are still debated [5]. This difficulty might be related to the fact that NT-proBNP values vary in association with demographic variables, treatment-related variables, and several medical conditions. For example, NT—proBNP concentrations are typically higher among elderly patients [6], women [7], and lower among individuals with significant obesity [8]. Additionally, renal impairment [9], low serum hemoglobin [6], and low systolic blood pressure (SBP) [10] have been associated with higher NT-proBNP concentrations. Attempts to determine cut-off scores need to also take into account that lack of compliance or withdrawal of treatment might impact serum NT-proBNP concentrations [11]. Consequently, NT-proBNP cut-off scores proposed to predict long-term outcomes after an episode of HF decompensation might vary for specific patient groups. Of note, most studies used pre-discharge rather than admission values, given that the former are less influenced by patients’ physical condition at admission and more influenced by the way symptoms of congestion responded to the treatment administered in the hospital [12].

Patients with HF and LVEF < 50% are at increased risk of long-term mortality [13]. Thus, the purpose of the present study was to examine if pre-discharge NT-proBNP values are predictive of mortality over a two-year period in a sample of Romanian patients with symptomatic HF and LVEF < 50%. Second, this study aimed to determine an effective threshold for NT-proBNP concentrations, above which the long-term risk of death is significantly increased. Third, the present study aimed to identify which variables may be associated with augmented NT-proBNP values (and implicitly to an elevated risk for long-term mortality).

## 2. Materials and Methods

### 2.1. Participant Selection

**Figure 1:**
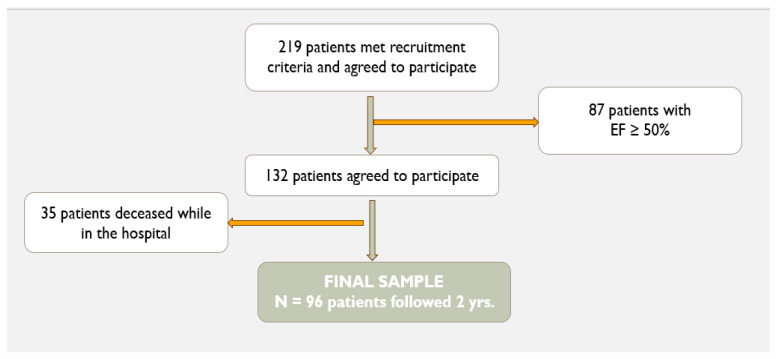
Study sample selection.

Participants were patients admitted to the Cardiology Clinic of the Elias Emergency University Hospital in Bucharest, Romania between March 2020 and October 2021. Initial inclusion criteria were: patients who presented to the hospital with signs and symptoms characteristic of HF (particularly acute dyspnea of cardiac origin), who had NT-proBNP at admission >250 pg/mL, and were able to comprehend the information regarding the study. Exclusion criteria were: patients with other conditions that could cause shortness of breath (e.g., pathological changes on the chest X-ray that led to the diagnosis of pneumonia or other lung diseases) or a diagnosis of chronic pulmonary diseases (asthma or COPD), or other conditions that could cause a rise in serum NT-proBNP concentrations (i.e., acute coronary syndromes, acute pulmonary embolism, pericardial constriction and obstructive hypertrophic cardiomyopathy, severe left heart valve disease, chronic kidney disease stage 4 or 5). This initial sample was reduced because 35 patients died in the hospital. Also, patients with preserved ejection fraction (i.e., LVEF ≥ 50%) (N = 87) were not included in the statistical analyses, given that this study focused on patients with LVEF < 50%. The final sample consisted of N = 96 patients who already had a diagnosis of HF with LVEF < 50%. Steps for participant selection are illustrated in Figure 1.

### 2.2. Design and Method

The present prospective study was conducted in accord with the declaration of Helsinki and was approved by the Ethics Committee of the Elias Emergency University Hospital—Bucharest, Romania (protocol number 3636 from 19 May 2021). Hospitalized patients who met the inclusion criteria were approached by one of the researchers who explained to them the purpose of the study and study procedures, discussed confidentiality issues, and let them know that the decision to participate or not would not influence in any way their treatment in the hospital. Patients who agreed to participate signed the informed consent form. All patients were evaluated from a clinical and biological standpoint (i.e., current symptoms, blood pressure and heart rate, and standard laboratory samples). In addition, we collected data regarding demographics, as well as associated comorbidities and at-home medication regimens. Patients received treatment according to guidelines. Just prior to their hospital discharge, blood samples were drawn once more, including serum NT-proBNP. Each patient received a medication prescription at discharge. Follow-up aimed to determine if patients were still alive or the date they were deceased, respectively. No patients were lost from follow-up, given that multiple attempts were made to contact patients by phone using the contact information they had provided at enrollment. Patient participants were registered as alive at the time of follow-up if the researchers were able to discuss with the patient or a close family member on the phone. In cases where patients could not be reached by phone (nobody answered, number not reachable, etc.), deaths were confirmed through hospital records or health insurance records.

### 2.3. Statistical Analyses

The software used to perform the statistical analyses for this study was SPSS (Statistical Package for the Social Sciences) version 17.0 (IBM SPSS Statistics, IBM Corp., Armonk, NY, USA). Group differences were evaluated using independent samples t tests for continuous variables, and chi-square (χ^2^) tests for discrete variables. In order to assess whether NT-proBNP values recorded at discharge significantly predict mortality over a two-year period, we used a Cox proportional hazards regression model. Second, a NT-proBNP cut-off score for risk of death was estimated based on the receiver operating characteristic (ROC) analysis and using the Youden J statistic to maximize the sensitivity and specificity [14]. With the purpose of validating this cut-off score, we also conducted second analyses on a sub-sample of 52 patients randomly selected from the study sample. This sub-sample maintained the same distribution of deceased versus alive patients at the end of the follow-up period, which was 1:2.3 patients. In order to examine specific variables that may be associated with NT-proBNP scores above the cut-off, we used univariate regressions with NT-proBNP concentration higher than the cut-off (yes/no) as the binary dependent variable and with the following predictors: age, systolic blood pressure at admission (SBP), presence vs. absence of ACE-I treatment, serum hemoglobin (HB), blood urea levels at admission, and serum platelet count at admission. Odds ratios were computed for the variables found to be significantly associated with NT-proBNP values above the established cut-off score. A multivariable model was also tested using a logistic regression with NT-proBNP score above/below the cut-off as dependent variable; the predictors tested in this model were the variables found to have a significant effect in univariate tests (i.e., age, serum hemoglobin, systolic blood pressure at admission, and lack of ACE-I treatment).

## 3. Results

### 3.1. Descriptive Statistics

The final sample used for statistical analyses was composed of 96 patients (53 male and 43 female) with HF and LVEF below 50% (median LVEF was 35%). Mean age was 74.8 ± 10.5 years old. Median NT-proBNP value at admission for the entire study sample was 8226 pg/mL and at discharge 7275 pg/mL. Patients were hospitalized for an average of 8.3 days (standard deviation = 5.28 days). Subgroup statistics for cardiovascular risk factors, treatments received, and biological markers are presented in Table 1.

### 3.2. Power Analysis

A post hoc power analysis was conducted using the G*Power version 3.1.9.7 [15] to determine the statistical power given our available sample size. A significance criterion of α = 0.05, an effect size d = 0.05, and a sample size N = 96 were used. The obtained power (1 − β error probability) = 0.67.

### 3.3. Prediction of Mortality from NT-proBNP Values

**Figure 2:**
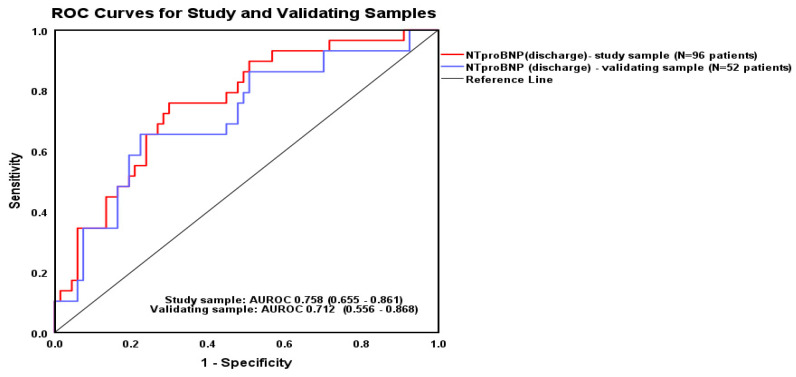
The ROC curves predicting the two-year mortality from NT-proBNP values at hospital discharge.

In our sample of 96 patients with HF, the Cox proportional hazards model with pre-discharge NT-proBNP concentrations predicted mortality at two years, AUC = 0.758 (95% CI [0.655–0.861], *p* 0.001). The binary regression equation used to assess risk of death based on NT-proBNP at discharge was: log OR [mortality at 2 years] = −1.96 + 0.096 * NT-proBNP (ng/mL). The prediction equation was also tested on a validation sub-sample (N = 52 patients), with AUC = 0.712 (95% CI [0.556–0.861], *p* 0.001). Thus, there was a great deal of overlap between the two models (AUROC in the study group 0.758 versus 0.712 in the validation cohort) and no significant difference between the two AUROCs (the Hanley and McNeil test was 0.54). The ROC curves for the total study sample and for the validation sub-sample are presented in Figure 2.

### 3.4. Cut-Off for NT-proBNP Values

**Figure 3:**
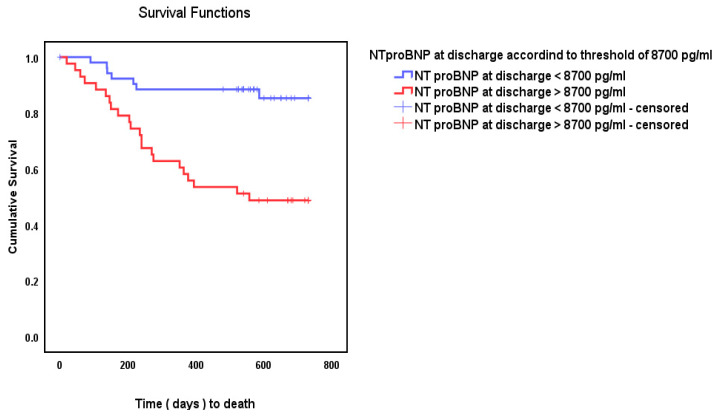
Kaplan–Meier cumulative survival using the cut-off score of 8700 pg/mL.

We tested several pre-discharge NT-proBNP cut-off scores predicting mortality at follow-up. The ROC curve and the curve coordinates table were consulted to determine the cut-off with the most favorable sensitivity and specificity. For the purpose of this study, the value of 8700 pg/mL was retained (see Table 2). Lower cut-offs were considered to have a too-high rate of false positives. Based on a Cox proportional hazards model, patients with NT-proBNP higher than 8700 pg/mL had a 4.6-fold increased chance of mortality (95% CI [2–10.8], *p* < 0.001) (Figure 3).

From among the 53 patients with NT-proBNP lower than the proposed cut-off score of 8700 pg/mL, 46 survived and 7 died. From among the 43 patients with NT-proBNP higher than 8700 pg/mL, 22 died and 21 survived (χ^2^ = 16.66, *p* < 0.001).

**Figure 4:**
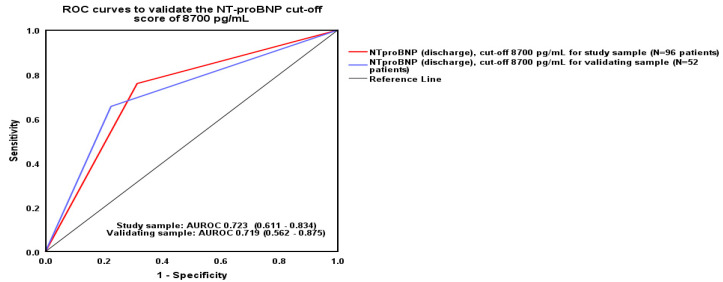
ROC curves for study and validation sample.

This cut-off score was validated through comparing the study sample against the validation sub-sample regarding their AUROCs—which were 0.723 and 0.719, respectively (Hanley & McNeil test significance value *p* = 0.92). Figure 4 depicts the ROC curves for the two groups of patients (i.e., study sample and validation sub-sample).

### 3.5. Predictors of NT-ProBNP Above the Threshold of 8700 Pg/mL

To determine what factors might be associated with NT-proBNP scores being higher than the cut-off of 8700 pg/mL, as a first step significant associations with demographic and medical variables were established (see Table 3). Those variables that rendered significant associations were further entered in univariate analyses, followed by multivariable tests.

Univariate regressions were conducted to determine predictors of NT-proBNP concentrations being higher than 8700 pg/mL. In each of these regression models, NT-proBNP above the cut-off score (yes/no) represented the binary outcome of interest and the predictors for each regression model were each one of the following variables: age, systolic blood pressure (SBP), presence versus absence of ACE-I treatment, and serum hemoglobin (HB) at admission. The findings are summarized in Table 4.

**Figure 5:**
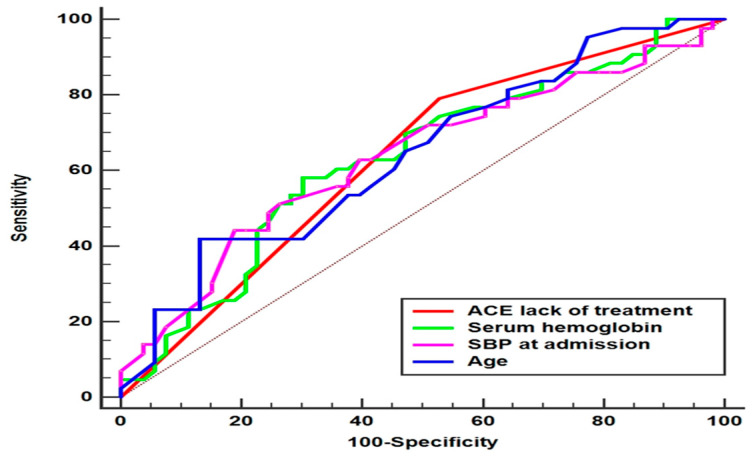
Prediction curves for NT-proBNP at discharge > 8700 pg/mL.

Survival analysis estimates were computed for the variables found to be predictors of NT-proBNP over 8700 pg/mL (i.e., age, SBP, no ACE-I treatment, and serum HB). Statistically significant results were obtained for age > 82 years old, OR 4.73 (95% CI [1.74–12.85], *p* = 0.002); systolic blood pressure (SBP) at admission ≤ 120 mmHg, OR 3.4 (95% CI [1.36–8.49], *p* = 0.009); lack of ACE-I treatment, OR 0.3 (95% CI [0.12–0.74], *p* = 0.009), and serum hemoglobin (HB) at admission ≤ 11.7 g/d, OR 3.2 (95% CI [1.38–7.46], *p* = 0.007). Figure 5 depicts the ROC curves for patients based on the variables.

A multivariable binary logistic regression was also conducted using the backward stepwise method, with NT-proBNP cut-off concentrations as the binary dependent variable (i.e., lower/higher than 8700 pg/mL). Multicollinearity between the four predictors (i.e., age, systolic blood pressure, ACE-I treatment, and serum hemoglobin) was not a concern, as the only significant correlation was age–serum hemoglobin, r(96) = −0.215, *p* = 0.035. The model summary indicated that Nagelkerke R^2^ = 0.281. Older age was significantly associated with higher chances of NT-proBNP concentrations over the cut-off, with OR = 1.07, 95% CI (1.01–1.12), *p* = 0.008 and back of ACE-I treatment was also associated with higher chances of NT-proBNP concentrations > 8700 pg/mL, OR = 0.26, 95% CI (0.09–0.72), *p* = 0.010, but hemoglobin and systolic blood pressure were not significant predictors of higher NT-proBNP concentrations in this multivariable model. The multivariable regression equation is: Log Odds Ratio (NT-proBNP > 8700 pg/mL) = −1.011 + 0.068 × age (years) − 1.339 × ACE-I administration − 0.139 × Hb (g/dL) − 0.016 × SBP (mmHg). Of note, if hemoglobin was conditionally removed from the prediction equation, systolic blood pressure approaches significance with OR = 0.984, 95% CI (0.96–1.00), *p* = 0.051.

## 4. Discussion

The present study aimed to determine if pre-discharge NT-proBNP concentrations may predict mortality over a period of two years among Romanian patients with HF and LVEF under 50%. First, we found that NT-proBNP at discharge was indeed an independent predictor of mortality at two years, and a cut-off value of 8700 pg/mL was found to predict a 4.6-fold increase the 2-year mortality risk with a 75.8% specificity and a 70.1% sensitivity.

Our proposed cut-off for NT-proBNP concentrations predicting 2-years mortality is higher than most other cut-off scores proposed by other published papers, and yet factors related to the study sample or to the research design might be associated with this difference. For example, a randomized study of patients with HF with reduced LVEF, revealed that regardless of baseline concentration, the adjustment of treatments so that NT-proBNP values drop below 1000 pg/mL was associated with a significant reduction in hospitalizations and mortality at 90 days [16]; yet, the cut-off in this study was estimated based on a patient population significantly younger than the one in the present study. Moreover, a lower NT-proBNP cut-off proposed as a predictor of 1-year mortality among patients with acute myocardial infarction was NT-proBNP level ≥ 1995 pg/mL [17]. However, while the sample of patients in this study was characterized by a mean age of 55.8 years and a preserved ventricular function with the mean LVEF being 51.8%, the sample of patients in our study had a mean age of 74.8 years and mean LVEF of 33.5%; patients with LVEF < 40%, as compared to those with LVEF > 50%, had a 4-times increased risk for all-cause one-year mortality [17]. Another cut-off proposed for NT-proBNP concentrations at admission was 5180 pg/mL (sensitivity, 68%; specificity, 72%) for predicting 76-day mortality [18]. However, while the median NT-proBNP concentration at admission in this study was 4639 pg/mL, for patients in our study the median admission NT-proBNP concentration was 8226 pg/mL. A study including a sample similar to ours (i.e., 99 patients with symptomatic HF, with a mean age of 71 years, with LVEF < 50% and a median NT-proBNP at admission of 6293.7 pg/mL) found that a NT-proBNP cut-off for predicting the 4-year survival was 2300 pg/mL (85.9% sensitivity and 39.1% specificity) [19]. However, this lower cut-off offered a lower specificity than the one from our study (39.1% versus 70.1% in our study). Significantly, findings from a meta-analysis involving patients admitted for acute decompensated HF suggested that cut-offs higher than 8700 pg/mL may be used. Specifically, the authors found that NT-proBNP levels between 5001 and 15,000 pg/mL at discharge were associated with 24% risk of death at 6 months after discharge and levels over 15,000 pg/mL had a mortality of 41% [20]. This finding is even more important as the median NT-proBNP at admission in this study was 6447 pg/mL (lower than the median value of our patient sample). Additionally, methodological factors possibly associated with the discrepancy between our proposed cut-off versus the ones proposed by other studies include the follow-up time interval (two years) and the fact that the vast majority of our patients could not benefit from the beneficial effects of SGLT2 inhibitors and ARNI as these medications were not yet covered by insurance for patients with HF in our country.

Another finding was that the likelihood of pre-discharge NT-proBNP being over the cut-off was higher for older patients, patients with low systolic blood pressure, those without ACE-I medication, and with low levels of serum hemoglobin (based on univariate tests). Age, systolic blood pressure, and ACE-I medication remained significant in the multivariable equation.

Regarding age, it is a well-documented fact that levels of NT-proBNP increase with age [21,22] and thus higher cut-off points need to be established for older patients. Thus, with the occasion of periodic evaluations of their patients with HF, community physicians need to pay particular attention to polypharmacy for co-morbidities, drug interactions, and medication tolerance issues among these patients. The association between ACE-I medication and NT-proBNP concentrations was not surprising, given the bradykinin-mediated anti-remodeling effect of this medication class (i.e., acts toward preventing ventricular dilatation and cardiac hypertrophy) [23]. ACE-I medication is a category IA indication for patients with HF according to guidelines [5,24] and thus its absence is expected to be associated with a more advanced disease stage and higher NT-proBNP concentrations. Indeed, hospitalized patients who continued ACE-I therapy experienced significantly lower mortality rates at 30 days, 90 days, and 1 year, as well as reduced 30-day readmission rates [25,26]. Thus, ACE-I treatment needs to be carefully evaluated and monitored with the occasion of patients’ medical check-ups, and potential side effects of these medications and/or dosage adjustments should be explored by generalists who monitor patients on the long-term. Serum hemoglobin is indicative of anemia—an undesirable common occurrence among patients with HF, with a multifactorial pathophysiology (iron deficiency, chronic inflammation, abnormal activation of the renin–angiotensin–aldosterone system, and/or abnormal levels of erythropoietin) [27]. Patients with anemia were three-times more likely to have NT-proBNP concentration over the age-specific cut-offs [28] and are at increased risk of mortality [27]. Consequently, this finding supports previous research despite that, due to logistic limitations, other serum tests (i.e., ferritin or transferrin) could not be assessed to further determine the nature of patients’ anemia. The association between low systolic blood pressure at admission and NT-proBNP > 8700 pg/mL suggests that patients with symptomatic HF and SBP under 120 mmHg might be at increased risk for unfavorable outcomes. A possible explanation is that lower blood pressure may be a sign of lower cardiac output, which is related to impaired ventricular function—thus a higher risk of death [6,29]. Indeed, HF with reduced ejection fraction might cause low blood pressure and other studies supported this result—especially for patients over 85 years [30]. Further research is needed to explore this association for particular age groups.

Notably, we did not find a significant association for several variables traditionally associated with higher NT-proBNP concentrations, such as gender (i.e., NT-proBNP concentrations being naturally higher among women) or chronic kidney disease. Also, while previous research suggested that NT-proBNP cut-off scores should be lower as BMI increases [8], in our study obesity was not found to be significantly associated with NT-proBNP; this finding is however debatable, as only 3 patients in our sample were classified as ‘obese’.

### 4.1. Strengths and Limitations

First, a strength of this study was the longitudinal design, which offers a valuable prognostic insight—especially given the longer follow-up of two years. Estimating HF risk over a longer time interval is more relevant for community follow-up as compared to the time period immediately after hospital discharge (which is often considered to maintain an ‘acute’ nature given the very high risk of recurrence). Another strength was that the study was conducted on Romanian patients with HF—a population with many needs but less studied. Third, we believe that an advantage was the inclusion of patients with HF with an ejection fraction below 50%, given that this is a population at higher risk of fatal outcomes.

A major limitation of this study was the small sample size, which led to a low statistical power (as indicated by the power analysis) and limited our ability to perform more complex analyses. A possible explanation may be that elderly patients in Romania may be reticent to participate in research studies; unfortunately, our limited resources did not allow provision of any incentives to our patient participants. Also, subgroup analyses based on other variables which might have been significantly associated with mortality (e.g., HF severity, co-morbidities, age groups, etc.) were not possible due to the small sample size available through the single center where this study was conducted.

Moreover, it was logistically impossible to record follow-up data regarding patients’ medications received at home, dosage adjustments, new diagnoses of co-morbidities, etc. Also, this study could not account for patients’ treatment adherence. Consequently, future studies are needed on larger sample sizes, which would stratify analyses based on LVEF groups and would include data regarding patients’ specific medical regiments, adherence to these regiments, as well as means for ambulatory/community healthcare services. Another limitation is that, due to the small number of patients, it was not feasible to delineate HF with LVEF < 40% versus 40–50% (mildly reduced EF). Yet another significant limitation is that the role of newer medications for HF (i.e., SGLT-2 inhibitors and ARNI) could not be examined since no patients were on SGLT-2 inhibitors and only 6 patients were on ARNI at the time this study occurred, due to the fact that in Romania these medicines became covered by insurance for patients with HF after the completion of this study. Lastly, participants were recruited in a single medical center; therefore, a multicenter, larger sample would have strengthened our findings.

### 4.2. Implications for Community Follow-Up

The findings from this longitudinal study have multiple implications regarding the long-term, community monitoring of patients with HF and reduced LVEF after being hospitalized for decompensated HF. Continuous monitoring of patients with HF through community health services has been associated with reduced re-hospitalizations [31], reduced mortality rates [32], and reduced healthcare expenditures [33]. First, an implication for clinical practice is the possibility to use the proposed cut-off score as part of a risk stratification model formulated prior to hospital discharge based on NT-proBNP levels, as well as on other risk factors (i.e., older age and lack of ACE-I treatment in particular, but also lower SBP and lower hemoglobin at admission). Such an individualized risk model, developed through effective communication between specialists and primary care physicians, would provide a comprehensive follow-up plan, including specifications regarding the frequency of medical check-ups and level of patient monitoring. Given the nature of their healthcare role, community physicians are those able to monitor patients with HF on a continuous basis—they have the ability to assess treatment effectiveness and potential side effects, and to adjust dosages as needed [34]. Additionally, for those patients who are at higher risk, it might be helpful to develop healthcare educational training materials to increase these patients’ levels of medical self-awareness (i.e., understand ‘alarm signs’ indicative of HF worsening), and also to have ‘safety plans’ in place (how and when is appropriate to immediately notify their first care provider versus specialist physicians, or access emergency medical services as needed). In the context of HF, patients’ ability to self-monitor parameters such as weight, blood pressure, heart rate, leg edema, or dyspnea has been associated with a reduction in negative HF-associated events because it allows prompt accessibility of healthcare services and adjustment of medical regimens [35].

## 5. Conclusions

Serum levels of NT-PROBNP may be used as tool for predicting long-term mortality among patients with symptomatic HF, with the goal of providing adequate monitoring to these patients with an increased focus on those deemed to be at increased risk. However, NT-PROBNP does not represent a fit-for-all assessment, and, consequently, it should be interpreted in an individualized manner, based on patient’ characteristics (i.e., age, LVEF, comorbidities), vital parameters (e.g., hemoglobin), as well as medication regimens (particularly ACE-I medication).

## Figures and Tables

**Table 1:** Descriptive statistics by subgroup (deceased vs. not deceased and NT-proBNP </≥ 8700 pg/mL).

	Alive N = 67					Deceased N = 29					*p*
	<8700 pg/mL N = 46 pts.		≥8700 pg/mL N = 21 pts.		*p*	<8700 pg/mL N = 7 pts.		≥8700 pg/mL N = 22 pts.		*p*	
Sex (female/male)	21/25		5/16		*0.110*	3/4		14/8		*0.403*	*0.080*
Risk factors											
Obesity	3		0		*0.317*	0		0		*-*	*-*
CKD	5		5		*0.156*	1		6		*0.444*	*0.382*
Diabetes	15		7		*0.583*	2		5		*0.556*	*0.473*
High BP	32		11		*0.139*	5		14		*0.541*	*1.000*
MI in the past	5		5		*0.156*	1		4		*0.653*	*0.767*
CHD	7		2		*0.417*	1		3		*0.692*	*1.000*
AFib	16		7		*0.568*	5		9		*0.166*	*0.254*
Stroke	6		1		*0.419*	1		2		*0.100*	*0.100*
Stent	0		3		0.028 *	1		0		*0.241*	*1.000*
Medication											
Diuretics (loop)	24		17		*0.022*	5		11		*0.292*	*0.653*
ACE-I	16		3		0.037 *	0		8		*0.075*	*0.811*
ARB	10		5		*0.542*	1		2		*0.579*	*0.255*
CCB	12		1		0.036 *	2		3		*0.347*	*1.000*
BB	29		16		*0.219*	5		14		*0.541*	*1.000*
Antithrombotic	21		11		*0.402*	3		12		*0.458*	*0.825*
Anticoagulant	15		8		*0.432*	6		8		0.031 *	*0.254*
Hypolipidemic	22		11		*0.467*	4		8		*0.295*	*0.512*
Digoxin	9		0		0.026 *	4		3		0.038 *	*0.237*
	*M*	*SD*	*M*	*SD*		*M*	*SD*	*M*	*SD*		
Age	71.7	10.2	73.5	7.8	*0.478*	75.8	13.5	82.4	8.8	*0.164*	<0.001 *
NT-proBNP_admission_	4201.9	2927.1	20,771.1	6782.3	<0.001 *	4719.0	2337.0	24,546.1	7641.7	<0.001 *	<0.001 *
NT-proBNP_discharge_	3269.4	2403.9	18,338.7	6616.3	<0.001 *	3942.4	1852.3	20,075.8	7374.4	<0.001*	<0.001 *
Hemoglobin_admission_	12.68	2.0	11.8	2.0	*0.108*	11.27	2.64	11.16	2.15	*0.917*	0.011 *
Leukocytes_admission_	11,512.8	4877.0	12,870.0	5370.0	*0.310*	9130.0	3279.4	9580.0	4060.2	*0.792*	0.021 *
Platelets	228,815.2	105,372.4	287,523.8	116,204.8	0.045 *	200,714.2	104,106.8	246,186.5	102,978.1	*0.319*	*0.622*
SBP_admission_	148.5	31.9	126.3	28.7	0.009 *	136.5	25.2	139.9	31.3	*0.800*	*0.727*
DBP_admission_	84.5	17.6	71.4	18.2	0.007 *	75.0	10.6	81.82	17.7	*0.347*	*0.948*
Heart rate_admission_	97.3	20.4	102.9	28.8	*0.367*	93.0	19.8	99.5	22.3	*0.496*	*0.821*
CRP_admission_	31.7	37.6	36.6	56.4	*0.748*	29.6	20.5	39.0	50.0	*0.761*	*0.777*
Creatinine_admission_	1.1	0.59	1.3	.63	*0.258*	1.0	0.24	1.38	1.04	*0.368*	*0.587*
Blood urea_admission_	73.8	116.7	73.0	41.2	*0.976*	46.8	22.6	76.0	39.7	*0.078*	*0.513*
Days hospitalized	9.04	6.67	7.38	3.71	*0.291*	6.0	2.0	8.36	3.52	*0.105*	*0.537*

Significant findings are marked *. CKD = chronic kidney disease; BP = blood pressure; MI = myocardial infarction; CHD = coronary heart disease; AFib = atrial fibrillation; ACE-I = angiotensin converting enzyme inhibitors; ARB = angiotensin receptors blocker; CCB = calcium channel blocker; SBP = systolic blood pressure; DBP = diastolic blood pressure; CRP = C-reactive protein.

**Table 2:** Psychometric indices for the NT-proBNP cut-off of 8700 pg/mL.

Cut-Off Value of 8700 pg/mL	Study Sample	Validation Sample
	*Youden’s Index p = 0.928*	
**Sensitivity (%)**	75.86 (56.5–89.7)	68.75 (41.3–89.0)
**Specificity (%)**	70.15 (57.7–80.7)	77.78 (60.8–89.9)
**PPV (%)**	11.8 (8.1–16.9)	14 (7.5–24.6)
**NPV (%)**	98.2 (96.6–99.1)	97.9 (95.7–99.0)
**Positive LR**	2.54 (1.7–3.9)	3.09 (1.5–6.2)
**Negative LR**	0.34 (0.2–0.7)	0.4 (0.2–0.8)

PPV = positive predictive value; NPV = negative predictive value; LR = likelihood ratio.

**Table 3:** Results from *t*-tests and chi-square tests for the associations between demographic and medical variables with the likelihood of NT-proBNP scores being over the cut-off of 8700 pg/mL.

	<8700 pg/mL N = 53 pts.		≥8700 pg/mL N = 43 pts.		
	Frequency (N)		Frequency (N)		*p*
Gender (male)	29		24		*0.914*
Obesity	3		0		*0.113*
CKD	6		11		*0.069*
MI in the past	6		9		*0.197*
CHD	8		5		*0.622*
AFib	21		16		*0.809*
Diuretics	30		23		*0.760*
**ACE-I**	**25**		**9**		0.008 *
ARB (sartans)	4		5		*0.495*
CCB	13		4		*0.052*
BB	30		27		*0.539*
Antithrombotics	30		20		*0.325*
Anticoagulants	22		22		*0.345*
Hypolipidemics	32		20		*0.175*
Digoxin	7		3		*0.320*
	Mean	SD	Mean	SD	*p*
**Age**	**72.26**	**10.71**	**77.93**	**9.36**	0.008 *
Creatine (mg/dL)	1.13	0.55	1.35	0.85	*0.124*
Urea (mg/dL)	70.26	109.27	74.56	40.06	*0.807*
**Hb (g/dL)**	**12.49**	**2.15**	**11.48**	**2.09**	0.022 *
Platelets (×10^3^/µL)	225,103	104,649	266,374	110,316	*0.064*
Leukocytes (cells/µL)	11,198	4742	11,186	4974	*0.991*
CRP (mg/L)	31.59	36.19	37.83	52.26	*0.603*
**Systolic BP (mmHg)**	**146.92**	**31.16**	**133.3**	**30.54**	0.034 *
Diastolic BP (mmHg)	83.28	17.13	76.74	18.54	*0.076*
Heart rate (beats/min)	96.79	20.21	101.21	25.51	*0.346*
LVEF	34.47	10.24	32.47	7.99	*0.299*

Significant findings are marked in bold and significant *p* values are marked *. CKD = chronic kidney disease; MI = myocardial infarction; CHD = coronary heart disease; AFib = atrial fibrillation; ACE-I = angiotensin converting enzyme inhibitors; ARB = angiotensin receptors blocker; CCB = calcium channel blocker; CRP = C-reactive protein; Hb = hemoglobin; LVEF = left ventricular ejection fraction.

**Table 4:** Univariate predictors of NT-proBNP above the cut-off score of 8700 pg/mL.

Predictors of NT-proBNP > 8700 pg/mL	Intercept (β0)	Intercept *p*-Value	Coefficient β1	β1 *p*-Value
Age	−4.496	0.008	0.057	0.010 *
SBP at admission ≤ 120 mmHg	1.918	0.066	−0.015	0.039 *
No ACE-I treatment	0.194	0.440	−1.210	0.009 *
HB ≤ 11.7 g/dL	2.499	0.042	−0.226	0.026 *

Significant *p* values are marked *.

## References

[1] Sinnenberg L., Givertz M.M. (2020). Acute Heart Failure. Trends Cardiovasc. Med..

[2] Arrigo M., Jessup M., Mullens W., Reza N., Shah A.M., Sliwa K., Mebazaa A. (2020). Acute Heart Failure. Nat. Rev. Dis. Primers.

[3] Jones N.R., Roalfe A.K., Adoki I., Hobbs F.D.R., Taylor C.J. (2019). Survival of Patients with Chronic Heart Failure in the Community: A Systematic Review and Meta-analysis. Eur. J. Heart Fail..

[4] Parlati A.L.M., Madaudo C., Nuzzi V., Manca P., Gentile P., Di Lisi D., Jordán-Ríos A., Shamsi A., Manzoni M., Sadler M. (2025). Biomarkers for Congestion in Heart Failure: State-of-the-Art and Future Directions. Card. Fail. Rev..

[5] McDonagh T.A., Metra M., Adamo M., Gardner R.S., Baumbach A., Böhm M., Burri H., Butler J., Čelutkienė J., Chioncel O. (2021). 2021 ESC Guidelines for the Diagnosis and Treatment of Acute and Chronic Heart Failure. Eur. Heart J..

[6] Couissi A., Haboub M., Hamady S., Ettachfini T., Habbal R. (2024). Predictors of Mortality in Heart Failure Patients with Reduced or Mildly Reduced Ejection Fraction: The CASABLANCA HF Study. Egypt. Heart J..

[7] Fuat A., Murphy J.J., Hungin A.P.S., Curry J., Mehrzad A.A., Hetherington A., Johnston J.I., Smellie W.S.A., Duffy V., Cawley P. (2006). The Diagnostic Accuracy and Utility of a B-Type Natriuretic Peptide Test in a Community Population of Patients with Suspected Heart Failure. Br. J. Gen. Pract..

[8] Vergaro G., Gentile F., Meems L.M.G., Aimo A., Januzzi J.L., Richards A.M., Lam C.S.P., Latini R., Staszewsky L., Anand I.S. (2021). NT-proBNP for Risk Prediction in Heart Failure. JACC Heart Fail..

[9] McCullough P.A., Duc P., Omland T., McCord J., Nowak R.M., Hollander J.E., Herrmann H.C., Steg P.G., Westheim A., Knudsen C.W. (2003). B-Type Natriuretic Peptide and Renal Function in the Diagnosis of Heart Failure: An Analysis from the Breathing Not Properly Multinational Study. Am. J. Kidney Dis..

[10] Lang X., Peng C., Zhang Y., Gao R., Zhao B., Li Y., Zhang Y. (2024). Correlation between Systolic Blood Pressure and Mortality in Heart Failure Patients with Hypertension. J. Hypertens..

[11] Gilstrap L.G., Fonarow G.C., Desai A.S., Liang L., Matsouaka R., DeVore A.D., Smith E.E., Heidenreich P., Hernandez A.F., Yancy C.W. (2017). Initiation, Continuation, or Withdrawal of Angiotensin-Converting Enzyme Inhibitors/Angiotensin Receptor Blockers and Outcomes in Patients Hospitalized with Heart Failure with Reduced Ejection Fraction. J. Am. Heart Assoc..

[12] O’Brien R.J., Squire I.B., Demme B., Davies J.E., Ng L.L. (2003). Pre-discharge, but Not Admission, Levels of NT-proBNP Predict Adverse Prognosis Following Acute LVF. Eur. J. Heart Fail..

[13] Furtado R.H.M., Juliasz M.G., Chiu F.Y.J., Bastos L.B.C., Dalcoquio T.F., Lima F.G., Rosa R., Caporrino C.A., Bertolin A., Genestreti P.R.R. (2023). Long-term Mortality after Acute Coronary Syndromes among Patients with Normal, Mildly Reduced, or Reduced Ejection Fraction. ESC Heart Fail..

[14] Youden W.J. (1950). Index for Rating Diagnostic Tests. Cancer.

[15] Faul F., Erdfelder E., Lang A.-G., Buchner A. (2007). G*Power 3: A Flexible Statistical Power Analysis Program for the Social, Behavioral, and Biomedical Sciences. Behav. Res. Methods.

[16] Januzzi J.L., Ahmad T., Mulder H., Coles A., Anstrom K.J., Adams K.F., Ezekowitz J.A., Fiuzat M., Houston-Miller N., Mark D.B. (2019). Natriuretic Peptide Response and Outcomes in Chronic Heart Failure with Reduced Ejection Fraction. J. Am. Coll. Cardiol..

[17] Tan S.S.N., Koh K.T., Fong A.Y.Y., Bujang M.A.B., Tiong L.L., Cham Y.L., Ho K.H., Tan C.T., Khaw C.S., Mohd Amin N.H. (2022). NT-proBNP Cut-off Values for Risk Stratification in Acute MI and Comparison with Other Risk Assessment Scores. J. Asian Pac. Soc. Cardiol..

[18] Januzzi J.L., Van Kimmenade R., Lainchbury J., Bayes-Genis A., Ordonez-Llanos J., Santalo-Bel M., Pinto Y.M., Richards M. (2006). NT-proBNP Testing for Diagnosis and Short-Term Prognosis in Acute Destabilized Heart Failure: An International Pooled Analysis of 1256 Patients. Eur. Heart J..

[19] Velibey Y., Golcuk Y., Golcuk B., Oray D., Atilla O.D., Colak A., Kurtulmus Y., Erbay A.R., Yilmaz A., Eren M. (2013). Determination of a Predictive Cutoff Value of NT-proBNP Testing for Long-Term Survival in ED Patients with Acute Heart Failure. Am. J. Emerg. Med..

[20] Salah K., Kok W.E., Eurlings L.W., Bettencourt P., Pimenta J.M., Metra M., Bayes-Genis A., Verdiani V., Bettari L., Lazzarini V. (2014). A Novel Discharge Risk Model for Patients Hospitalised for Acute Decompensated Heart Failure Incorporating N-Terminal pro-B-Type Natriuretic Peptide Levels: A European coLlaboration on Acute decompeNsated Heart Failure: ÉLAN-HF Score. Heart.

[21] Hildebrandt P., Collinson P.O., Doughty R.N., Fuat A., Gaze D.C., Gustafsson F., Januzzi J., Rosenberg J., Senior R., Richards M. (2010). Age-Dependent Values of N-Terminal pro-B-Type Natriuretic Peptide Are Superior to a Single Cut-Point for Ruling out Suspected Systolic Dysfunction in Primary Care. Eur. Heart J..

[22] Collerton J., Kingston A., Yousaf F., Davies K., Kenny A., Neely D., Martin-Ruiz C., MacGowan G., Robinson L., Kirkwood T.B. (2014). Utility of NT-proBNP as a Rule-out Test for Left Ventricular Dysfunction in Very Old People with Limiting Dyspnoea: The Newcastle 85+ Study. BMC Cardiovasc. Disord..

[23] Remme W.J. (1997). Bradykinin-Mediated Cardiovascular Protective Actions of ACE Inhibitors: A New Dimension in Anti-Ischaemic Therapy?. Drugs.

[24] Heidenreich P.A., Bozkurt B., Aguilar D., Allen L.A., Byun J.J., Colvin M.M., Deswal A., Drazner M.H., Dunlay S.M., Evers L.R. (2022). 2022 AHA/ACC/HFSA Guideline for the Management of Heart Failure: A Report of the American College of Cardiology/American Heart Association Joint Committee on Clinical Practice Guidelines. Circulation.

[25] Krantz M.J., Ambardekar A.V., Kaltenbach L., Hernandez A.F., Heidenreich P.A., Fonarow G.C. (2011). Patterns and Predictors of Evidence-Based Medication Continuation Among Hospitalized Heart Failure Patients (from Get with the Guidelines–Heart Failure). Am. J. Cardiol..

[26] Pascual-Figal D., Bayes-Genis A. (2024). Looking for the Ideal Medication for Heart Failure with Reduced Ejection Fraction: A Narrative Review. Front. Cardiovasc. Med..

[27] Siddiqui S.W., Ashok T., Patni N., Fatima M., Lamis A., Anne K.K. (2022). Anemia and Heart Failure: A Narrative Review. Cureus.

[28] Willis M.S., Lee E.S., Grenache D.G. (2005). Effect of Anemia on Plasma Concentrations of NT-proBNP. Clin. Chim. Acta.

[29] Barlera S., Tavazzi L., Franzosi M.G., Marchioli R., Raimondi E., Masson S., Urso R., Lucci D., Nicolosi G.L., Maggioni A.P. (2013). Predictors of Mortality in 6975 Patients with Chronic Heart Failure in the Gruppo Italiano per Lo Studio Della Streptochinasi Nell’Infarto Miocardico-Heart Failure Trial: Proposal for a Nomogram. Circ. Heart Fail..

[30] Poortvliet R.K.E., Blom J.W., De Craen A.J.M., Mooijaart S.P., Westendorp R.G.J., Assendelft W.J.J., Gussekloo J., De Ruijter W. (2013). Low Blood Pressure Predicts Increased Mortality in Very Old Age Even without Heart Failure: The Leiden 85-plus Study. Eur. J. Heart Fail..

[31] Liu S., Graves N., Ma C., Pan J., Xie Y., Lee S.Y.A., Senanayake S., Kularatna S. (2025). Preventability of Readmissions for Patients with Heart Failure—A Scoping Review. Heart Lung.

[32] Savage H.O., McBeath K., Hogan J., MacKay-Thomas L., Anderson L., Smith A., Bateman J., Brooks P., Bayes-Genis A., Vest A. (2024). The 25in25 Initiative: A Novel Transformative Project to Reduce Mortality Due to Heart Failure by 25% in the next 25 Years. Eur. J. Heart Fail..

[33] Galbreath A.D., Krasuski R.A., Smith B., Stajduhar K.C., Kwan M.D., Ellis R., Freeman G.L. (2004). Long-Term Healthcare and Cost Outcomes of Disease Management in a Large, Randomized, Community-Based Population with Heart Failure. Circulation.

[34] Teleanu I.C., Mîrșu-Păun A., Bejan C.G., Stănescu A.-M.A. (2025). NT-proBNP for Heart Failure Screening in Primary Care in an Eastern European Country: What We Know and Proposed Steps. Epidemiologia.

[35] Desai A.S., Stevenson L.W. (2012). Rehospitalization for Heart Failure: Predict or Prevent?. Circulation.

